# A Victorivirus and Two Novel Mitoviruses Co-Infected the Plant Pathogen *Nigrospora oryzae*

**DOI:** 10.3390/v11010083

**Published:** 2019-01-19

**Authors:** Hong Liu, Rui Liu, Chang Xin Li, Hui Wang, Hong Jian Zhu, Bi Da Gao, Qian Zhou, Jie Zhong

**Affiliations:** Hunan Provincial Key Laboratory for Biology and Control of Plant Diseases and Insect Pests, Hunan Agricultural University, Changsha 410128, China; 15526497045@163.com (H.L.); LR3131560@163.com (R.L.); changxinli1998@sina.com (C.X.L.); wh18597847174@163.com (H.W.); hongjian62@163.com (H.J.Z.); bdgao@aliyun.com (B.D.G.)

**Keywords:** mycovirus, *Nigrospora oryzae*, victotivirus, mitovirus

## Abstract

Three dsRNAs, in sizes of approximately 2.5–5 kbp, were detected in the plant pathogenic fungus *Nigrospora oryzae* strain CS-7.5-4. Genomic analysis showed that the 5.0 kb dsRNA was a victorivirus named as *Nigrospora oryzae* victorivirus 2 (NoRV2). The genome of NoRV2 was 5166 bp in length containing two overlapping open reading frames (ORFs), ORF1 and ORF2. ORF1 was deduced to encode a coat protein (CP) showing homology to the CPs of viruses belonging to the Totiviridae family. The stop codon of ORF1 and the start codon of ORF2 were overlapped by the tetranucleotide sequence AUGA. ORF2 was predicted to encode an RNA-dependent RNA polymerase (RdRp), which was highly similar to the RdRps of victoriviruses. Virus-like particle examination demonstrated that the genome of NoRV2 was solely encapsidated by viral particles with a diameter of approximately 35 nm. The other two dsRNAs that were less than 3.0 kb were predicted to be the genomes of two mitoviruses, named as *Nigrospora oryzae* mitovirus 1 (NoMV1) and *Nigrospora oryzae* mitovirus 2 (NoMV2). Both NoMV1 and NoMV2 were A-U rich and with lengths of 2865 and 2507 bp, respectively. Mitochondrial codon usage inferred that each of the two mitoviruses contains a major large ORF encoding a mitoviral RdRp. Horizontal transfer experiments showed that the NoMV1 and NoMV2 could be cotransmitted horizontally via hyphal contact to other virus-free *N. oryzae* strains and causes phenotypic change to the recipient, such as an increase in growth rate. This is the first report of mitoviruses in *N. oryzae*.

## 1. Introduction

Fungal viruses, also called mycoviruses, are widespread in diverse fungal species. In general, most mycoviruses cause only cryptic infections, and their fungal hosts show no symptoms [1,2]. However, some mycoviruses can cause hypovirulence to their hosts, leading to visible, debilitating symptoms. These viruses were considered to be potential biocontrol agents against plant pathogenic fungi, as has been well exemplified by the Cryphonectria hypovirus 1 (CHV1) in *Cryphonectria parasitica*, successfully used to control chestnut blight in Europe [3]. Therefore, the discovery and identification of more mycoviruses can provide materials for biological control of fungal diseases. In general, mycoviruses contain either double-stranded (ds) RNA or positive single-stranded (+ss) RNA genomes. However, several other mycoviruses with their genomes composed of DNA or negative-sense ssRNA have also been reported in plant pathogenic fungus *Sclerotinia sclerotiorum* [4,5]. Based on current virus taxonomy, dsRNA mycoviruses are ascribed to seven families (*Partitiviridae*, *Reoviridae*, *Megabirnaviridae*, *Totiviridae*, *Chrysoviridae*, *Endornaviridae*, and *Quadriviridae*) [6]. With the discovery of other more dsRNA viral species that could not be assigned to any established genus or family, the taxonomy of mycoviruses was continually redefined [2,7,8,9].

Viruses in the Totiviridae family are divided into five genera: *Totivirus*, *Giardiavirus*, *Victorivirus*, *Leishmaniavirus*, and *Trichomonasvirus* (https://talk.ictvonline.org/taxonomy/). Members of the genus *Victorivirus* infect filamentous fungi [10,11]. The genomes of victorivirus, encased by partical virions of 30–40 nm in diameter, are 4.6–7.0 kb in length and contain two partially overlapping or contiguous open reading frames (ORFs), encoding putative proteins of CP and RdRp in the 5′- and 3′-proximal, respectively [9,12]. The downstream RdRp is expressed from the upstream CP using a coupled termination/reinitiation mechanism by an AUGA motif containing the stop codon of CP and the initiation codon of RdRp [13,14]. The +ssRNA mycoviruses are classified into at least seven families, including Alphaflexiviridae, Barnaviride, Gammaflexiviridae, Hypoviridae, and Narnaviridae [15]. Viruses in the Narnaviridae family are divided into genera *Narnavirus* and *Mitovirus* [16]. In general, mitoviruses are located in mitochondria and infect filamentous fungi, but they have also recently been found in the human pathogen *Aspergillus fumigatus* [17]. Members of the genus *Narnavirus* are present in protoplasm and infect yeasts, while putative members of the genus *Narnavirus* have been found in filamentous fungi as well [18]. The narnaviruses were considered as the simplest naked mycoviruses as their genome contains only one large ORF that encodes a mitoviral RdRp [1,15,16].

*Nigrospora oryzae* is a weak parasite plant pathogenic fungus that has a broad host range of over 300 plant species (https://nt.ars-grin.gov/fungaldatabases/). This fungus always causes leaf spot and rot diseases, which are responsible for yield losses of many economically important crops, including rice (*Oryza sativa*), wheat (*Triticum aestivum*), maize (*Zea mays*), sorghum (*Sorghum bicolor*), cotton (*Gossypium hirsutum*), aloe (*Aloe vera*), and kiwifruit (*Actinidia Chinensis*) [19,20,21,22]. At present, a few mycoviruses have been characterized from *N. oryzae*, including a novel partitiviruses [23] and a fusarivirus [24].

In this study, we report the isolation and molecular characteristic of three mycoviruses co-infecting the *N. oryzae*, which designated *Nigrospora oryzae* victorivirus 2 (NoRV2), *Nigrospora oryzae* mitovirus 1, and *Nigrospora oryzae* mitovirus 2. Genomic organization, phylogenetic trees, and viral particles morphology were analyzed for virus identification.

## 2. Materials and Methods

### 2.1. Fungal Isolation and Identification

*N. oryzae* strains CS-7.5-4 and CS-7.5-8 were isolated by tissue isolation from the diseased rice leaves collected in the same field. Fungi identity were identified by amplification and sequencing of the rDNA internal transcribed spacer (ITS), translation elongation factor 1-α (EF-1α), and β-tubulin genes. The ITS, β-tubulin, and EF-1α genes of the two strains were identical.

### 2.2. dsRNA Extraction and Purification

Strain CS-7.5-4 was cultured in PD broth via shaking (180 rpm) at 28 °C for 7 days. Subsequently, the mycelia were harvested by filtration with sterile filter paper. The dsRNA was isolated from mycelium using the CF11 cellulose affinity chromatography method as described by Morris and Dodds, with slight modifications [25]. The extractions were treated with DNase I and S1 nuclease at 37 °C for 40 min (TaKaRa, Dalian, China) to eliminate possible contaminating DNA and ssRNA, electrophoresed on a 1% agarose gel and then visualized under an UV trans-illuminator after being stained with GoldView. Each of the dsRNA segments was separately purified with a gel extraction kit (TaKaRa, Dalian, China), dissolved in DEPC-treated water, and stored at −20 °C until use.

### 2.3. cDNA Synthesis, Molecular Cloning, and Sequencing

Full-length cDNA cloning and sequencing of the purified dsRNA were performed using the methods as previously described with slight modifications [26]. Purified dsRNA were used as the templates for reverse transcription and cDNA library synthesis using random hexanucleotide primers and reverse transcriptase. All the amplified cDNA products were cloned into a T/A cloning vector, pMD18-T (TaKaRa, Dalian, China). The recombinant vectors were transformed into competent *Escherichia coli* DH5α cells and then selected and sequenced. Gaps that were not covered by the cDNA library in the initial round of sequencing were filled by RT-PCR amplification using specific primers designed based on the obtained cDNA sequences flanking the gaps. To clone the ends of the dsRNA, adapter ligation and amplification were carried out as described previously [27]. To ensure the sequence accuracy, each nucleotide of the dsRNA was sequenced at least three times. The final contigs were assembled and deposited in the GenBank database.

### 2.4. Sequence Analyses

A homology search was performed against the National Center for Biotechnology Information (NCBI) database (http://www.ncbi.nlm.nih.gov/genomes) using the BLASTp program. ORFs were deduced and translated by the ORF finder on the NCBI website. Sequences analysis and multiple alignments were conducted using DNAMAN and ClustalX [28]. On the basis of sequence alignment, phylogenetic trees were constructed using the NJ method by MEGA 6, with a bootstrap test of 1000 replicates [29]. Potential secondary structures of the 5 and 3 terminal sequences were predicted, and the free energy (G) was estimated using mfold (http://mfold.rna.albany.edu/?q=DINAMelt/Quickfold).

### 2.5. Extraction of Viral Particles and SDS-PAGE

Mycelia were cultured in liquid PD broth. About 30 g of mycelia was collected and ground into a fine powder in liquid nitrogen and then suspended in 50 mL of 0.1 M sodium-phosphate buffer (pH 7.0). The suspension was separated by centrifugation at 10,000 g for 30 min with 50% (v/v) chloroform and n-butanol (1:1). The resultant supernatant was adjusted to 8% PEG6000 and 1% NaCl and then shaking at 4 °C overnight. The suspension was centrifuged at 10,000× *g* for 30 min, layered onto 20% sucrose, and then ultra-centrifuged at 100,000× *g* for 3 h. The virus particle was suspended in 100 µL of 0.01 M sodium-phosphate buffer (pH 7.0) and then used for SDS-PAGE gel (8%) analysis and TEM observation. All processes were performed according to that described previously [30,31].

### 2.6. Horizontal Transmission of the Associated dsRNAs in N. oryzae

Horizontal transmission experiments were carried out using the pairing culture technique as previously described [32]. The dsRNAs-containing *N. oryzae* strain CS-7.5-4 was used as the donor, and a virus-free strain CS-7.5-8 was used as recipient. The transmission for the recipient strain was repeated three times. Mycelial plugs of the donor and recipient combination (CS-7.5-4/CS-7.5-8) were individually cultured 1 cm apart on PDA dish. Mycelial agar plugs were taken from the edge of each colony of the recipient (CS-7.5-8) to obtain recipient derivative isolates. All derivative isolates were analyzed for the presence of three mycoviruses via dsRNAs extraction and RT-PCR using primer pairs designed according to the genome sequences of NoRV2, NoMV1, and NoMV2 (Table S1). A derivative isolate, named CS-7.5-8-V, was selected for biological comparison, including colony morphology and mycelial growth rate, to their progenitor.

### 2.7. Extraction of the Total RNA and RT-PCR Detection of the Associated dsRNAs

Total RNA was extracted from five-day-old mycelia of each strain using Transzol up (TransGen Biotech, Beijing, China), treated by DNase I to remove DNA contamination. Next, the extract was used as a template for reverse transcription to synthesize cDNA using TransScript^®^ Reverse Transcriptase (TransGen Biotech) with the Random primer. Finally, the cDNA was used as template in PCR for amplification of the three dsRNAs with specific primers. The actin gene was used as an internal control in the RT-PCR detection.

## 3. Results

### 3.1. dsRNA Segments Isolated from Strain CS-7.5-4 of N. oryzae

**Figure 1:**
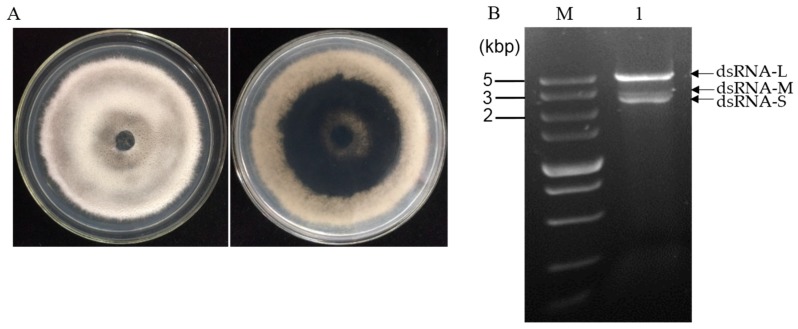
(**A**) Colony morphology of strain CS-7.5-4 after five days of culture on PDA at 28 °C in dark; (**B**) agarose gel electrophoresis of dsRNAs extracted from mycelium of strain CS-7.5-4. The extracted nucleic acids were treated with DNase 1 and S1 nuclease at 37 °C for 40 min and electrophoresed in a 1% agarose gel. Lane M, DNA marker (5 kb ladder, TaKaRa); Lane 1, dsRNA sample extracted from the *N. oryzae* strain CS-7.5-4.

*N. oryzae* strain CS-7.5-4 (Figure 1A) was isolated from a typical diseased rice leaf and identified by morphological data and amplification of the internal transcribed spacer region (ITS) of rDNA and the elongation factor 1-alpha (TEF1-α) gene. The dsRNAs were extracted from mycelial mass of the strain CS-7.5-4 using the CF-11 cellulose chromatography method and then treated by DNase I and S1 nuclease. Using a 1% agarose gel electrophoresis, a clear band of approximately 5 kb and two bands of approximately 3 kb (dsRNA-L, M, S) were observed (Figure 1B). The dsRNA-nature of these extracts were confirmed by treatments of DNase I and S1 nuclease, which could digest any potential contaminated DNA and ssRNA molecules. The three dsRNA segments were separately agarose gel-purified and subjected to cDNA cloning.

### 3.2. Genome Sequence Analysis of the dsRNA-L in Strain CS-7.5-4

To better understand the potential genetic information, the complete cDNA sequences of the three dsRNA segments were determined by conventional random priming cDNA synthesis, RT-PCR, and RACE cloning.

**Figure 2:**
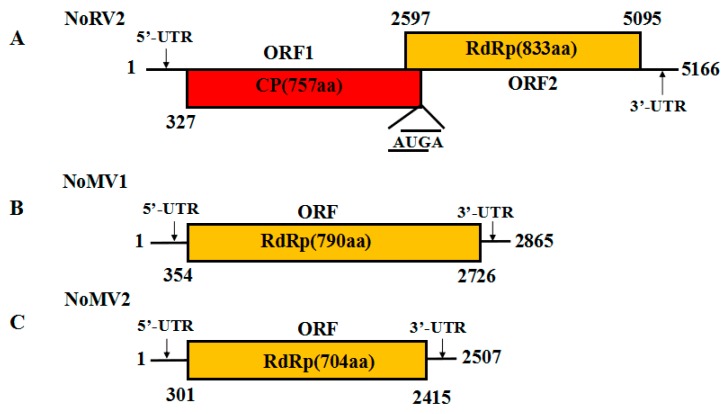
(**A**) Schematic representation of the genetic organization of NoRV2; the coding strand of the NoRV2 was 5166 bp long and predicted to contain two large ORFs, encoding a coat protein (CP, red) of 757 aa and an RNA-dependent RNA polymerase (RdRp, yellow) of 833 aa. (**B**,**C**) Schematic representation of the genetic organization of NoMV1 and NoMV2. The genome lengths of the NoMV1 and NoMV2 were 2865 and 2507 bp, respectively. Each of the two viruses contains one large ORF encoding mitoviral RdRps of 790 aa and 704 aa, respectively.

The complete genome sequence of the 5.0 kb dsRNA-L was determined and submitted to GenBank with accession No. MH823900. Sequence analysis showed that the dsRNA-L was 5166 bp long, with a G+C content of 59.9%. The 5′-untranslated region (UTR) and 3′-UTR were 326 and 71 bp, respectively. Two overlapping ORFs (ORF1 and ORF2) were detected on the positive strand of the dsRNA-L, with the ORF1 stop codon overlaps with the ORF2 start codon by a tetranucleotide sequence AUGA at nt positons 2596–2600. ORF1 was predicted to encode a protein of 757 amino acids (aa) residues with a calculated molecular mass of 79.8 kDa, while ORF2 potentially encoded a 91.1 kDa protein composed of 833 aa residues. The BLASTp search showed that the proteins encoded by both ORF1 and ORF2 were homologous to the CPs and RdRps of victoriviruses, particularly to that of *Helminthosporium victoriae* virus 190S (HvV190S) and *Bipolaris maydis* victorivirus 1 (BmV1) (Table 1). An H-type pseudoknot structure was predicted upstream of the tetranucleotide sequence AUGA, which was supposed to be necessary for reinitiation of the downstream viral RdRp translation, as has been elucidated in other victoriviruses such as HvV190S [14]. Based on the conserved domain database search and multiple protein alignment, a conserved viral RdRp domain (pfam02123, RdRP_4) was detected in the ORF2-encoded protein of the dsRNA-L, which contained eight conserved motifs that are characteristic of RdRps of dsRNA mycovirus (Figure S1). Therefore, we propose the 5.0 kb dsRNA-L to be the genome of a new species of victorivirus, which we named *Nigrospora oryzae* victorivirus 2 (NoRV2). The genetic organization of NoRV2 is shown in Figure 2A.

### 3.3. Genome Sequence Analysis of the dsRNA-M, S in Strain CS-7.5-4

The full-length cDNA sequences of the dsRNA-M, S were obtained and submitted to the GenBank with accession No. MH823901 and MH823902, respectively. Subsequent sequences analysis showed that they were the genomes of two mitoviruses, which we named as *Nigrospora oryzae* mitovirus 1 (NoMV1) and *Nigrospora oryzae* mitovirus 2 (NoMV2), respectively. The genetic organization of NoMV1 and NoMV2 is shown in Figure 2B,C.

The genome sequence of NoMV1 was 2865 bp in length with a rich A+U content of 63.6%. Using a fungal mitochondrial codon usage in ORF finder program, NoMV1 was predicted to contain a single ORF (positions 354–2726) flanked by the 5′- and 3′-UTRs of 353 and 139 bp, respectively. The ORF encoded an 88.2 kDa protein with 790 aa residues that was most closely related to the RdRp of soybean leaf-associated mitovirus 5 (GenBank accession No. ALM62240), with an aa identity of 41.3%. Compared to NoMV1, NoMV2 has a smaller genome of 2583 bp with an A + U content of 67.01%. The lengths of the 5′- and 3′-UTRs of the (+) strand were 300 and 92 bp, respectively. NoMV2 also possessed a large ORF encoding an 81.3 kDa protein of 704 aa residues when predicted using the mitochondrial translation table. A BLASTp search of the protein showed a 42.3% identity with respect to the RdRp sequences of *Alternaria arborescens* mitovirus 1 (GenBank accession No. YP 009270635) (Table 2).

**Figure 3:**
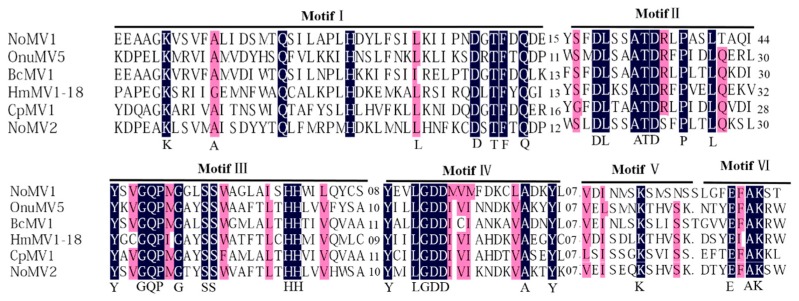
Multiple alignment of the deduced amino acid (aa) sequence of the RdRp encoded by NoMV1, NoMV2, and other related mitoviruses (OnuMV5, BcMV1, HmMV1-18, and CpMV1). Conserved motifs were indicated as I–VI above the lines. Shading: black: 100%; pink: >80% conservation.

A conserved domain search and multiple alignments based on viral RdRps of NoMV1, NoMV2, and other similar mitoviruses showed the presence of typical motifs (I–IV) that are characteristic of the mitoviral RdRp domain superfamily (pfam05919) (Figure 3) [33].

### 3.4. Predicted Secondary Structures of the 5′-UTRs and 3′-UTRs for the NoMV1 and NoMV2

Potential secondary structures of the 5′- and 3′-UTRs of NoMV1 and NoMV2 were predicted using mfold [34]. The 5′-UTRs and 3′-UTRs of NoMV1 could be folded into stem-loop structures at positions of 1–32 and 2830–2865 with ΔG values of –10.54 and –9.91 kcal/mol, respectively. Since the 5′- and 3′-UTRs of NoMV1 lacked inverted complementarity, they could not be folded into stable panhandle secondary structure. As for NoMV2, stem-loops were predicted at the 5′- (Positions 1–42) and 3′-terminal sequence (Positions 2416–2507), with ΔG values of −23.54 and −43.46 kcal/mol, respectively. A panhandle structure with the ΔG value of −27.80 kcal/mol was predicted, which was formed by the inverted complimentary sequences at 5′ and 3′ ends of the NoMV2 genome (Figure S2).

### 3.5. Phylogenetic Analysis of These Viruses Based on RdRps

**Figure 4:**
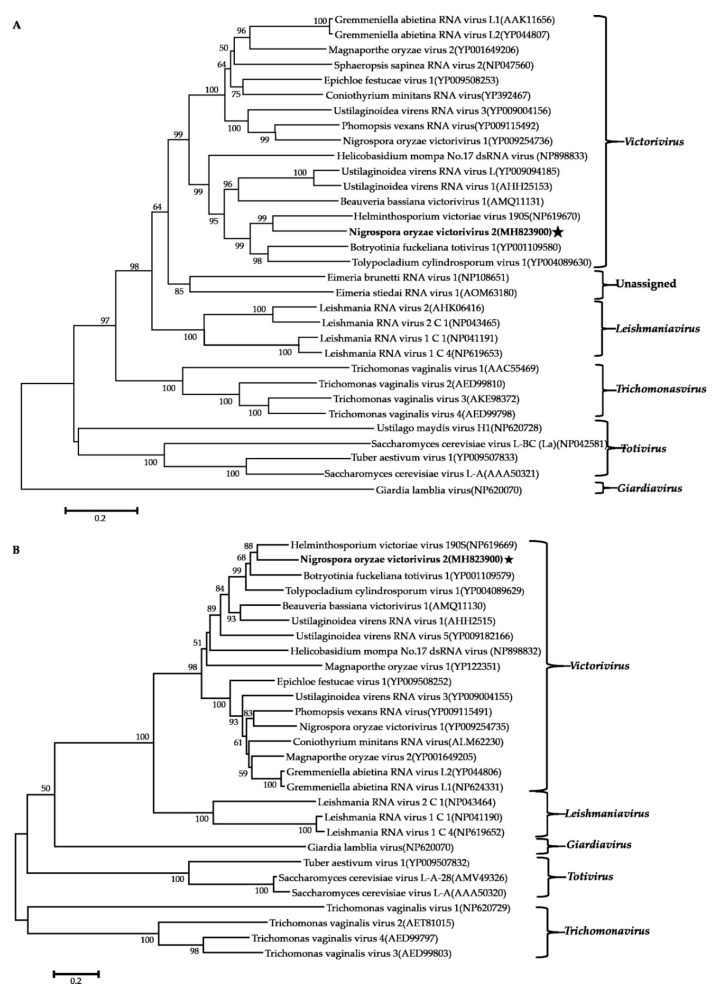
Phylogenetic analysis of NoRV2. Phylogenetic trees were constructed based on the RdRp (**A**) and CP (**B**) of NoRV2 and other selected viruses belonging to the family Totiviridae. Phylogenetic trees were constructed using neighbor-joining method, with 1000 bootstrap replicates by MEGA 6.0 software [29]. The bootstrap support values (%) were indicated proximal to nodes. The scale bar represents a genetic distance of 0.2 amino acid substitutions per site. The NoRV2 was indicated by black asterisk.

**Figure 5:**
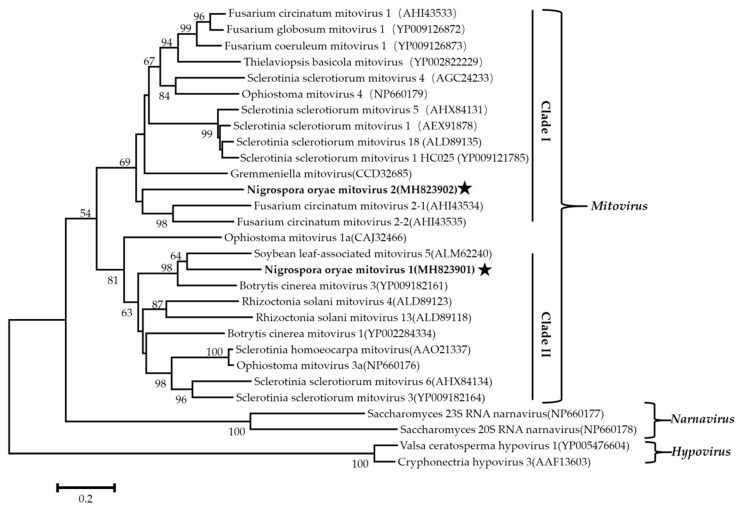
The phylogenetic tree was constructed based on the RdRps of NoMV1, NoMV2, and other narnaviruses using the neighbor-joining method, with 1000 bootstrap replicates by MEGA 6.0 software. The bootstrap values (%) supporting the branches are indicated at the nodes. The scale bar represents a genetic distance of 0.2 amino acid substitutions per site. The NoMV1 and NoMV2 were indicated by black asterisk.

Phylogenetic analysis using the neighbor-joining (NJ) method based on aa sequences of the viral RdRps was performed and revealed that the NoRV2 was clustered together with the HvV190S within the victorivirus clade that is distinct from members of the genera *Leishmaniavirus*, *Trichomonasvirus*, *Totivirus*, and *Giardiavirus* (Figure 4A). In addition, an NJ phylogenetic tree based on the aa sequences of the CP was also generated showing a topology similar to that of the RdRp-based tree (Figure 4B), thus supporting the phylogenetic status of the NoRV2. According to the genome organization and the phylogenetic analysis of the RdRp and CP, NoRV2 was proposed to be a new member of the genus *Victorivirus* in the Totiviridae family. To determine the evolutionary relationship between NoMV1, NoMV2, and other mitoviruses, phylogenic analysis was performed based on the RdRp aa sequences of NoMV1, NoMV2, and other selected viruses, including the mitoviruses, hypoviruses, and narnaviruses. Results revealed that NoMV1 and NoMV2 were clustered with the *Mitoviruses*, which divided into two clades with NoMV2 in Clade I and NoMV1 in Clade II (Figure 5). Based on genome organization and phylogenic analysis, the NoMV1 and NoMV2 identified here represent two novel species within the genus *Mitovirus*.

### 3.6. Purification of Viral Particles from Strain CS-7.5-4

**Figure 6:**
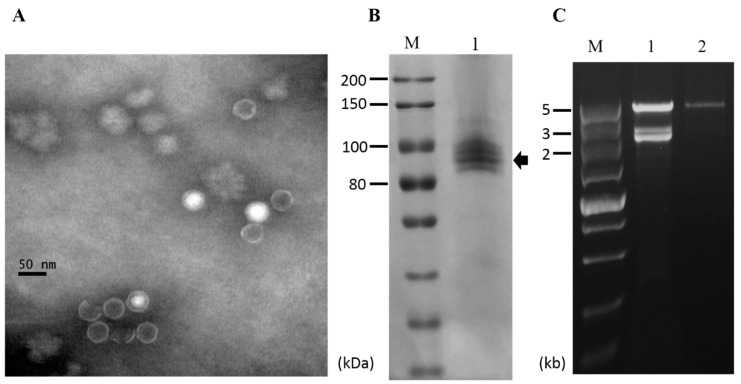
Virus particles of NoRV2. (**A**) Electron micrograph of viral particles. The viral particles were uranyl acetate-stained and observed on a transmission electron microscope. The scale bar indicates 50 nm. (**B**) Protein components of virus particles analyzed by an 8% SDS-PAGE gel-electrophoresis; Lane M, Protein marker (200 kDa ladder, TransGen); Lane 1, the viral particles purified from the *N. oryzae* strain CS-7.5-4. (**C**) dsRNA extraction from the viral particles. The extracted nucleic acids were treated with DNase 1 and S1 nuclease as described above and electrophoresed in a 1% agarose gel. Lane M, DNA marker (5kb ladder, TaKaRa). Lane 1, dsRNA isolated from mycelia of the CS-7.5-4; Lane 2, dsRNA isolated from viral particles.

To determine whether the viruses infecting strain CS-7.5-4 were encapsidated, virus particles were purified via ultracentrifugation. Transmission electron microscope (TEM) examination revealed the presence of isometric virus-like particles with a diameter of approximately 35 nm, resembling those reported for viruses in the Totiviridae family (Figure 6A). SDS-PAGE analysis of the virus particle preparations showed four major bands in calculative molecular weight of 80–100 kDa, which might include the coat protein encoded by NoRNV2 (Figure 6B). When extracted from the virus particle preparations, the dsRNA profile showed only as a single large 5 kb dsRNA segment which was distinguishable when compared to nucleic acids extracted directly from mycelia of the same strain CS-7.5-4 (Figure 6C). The result indicated that the virus-like particles was the viral capsids packaging only the genome RNA of NoRNV2.

### 3.7. Horizontal Transmission of the Associated dsRNAs

**Figure 7:**
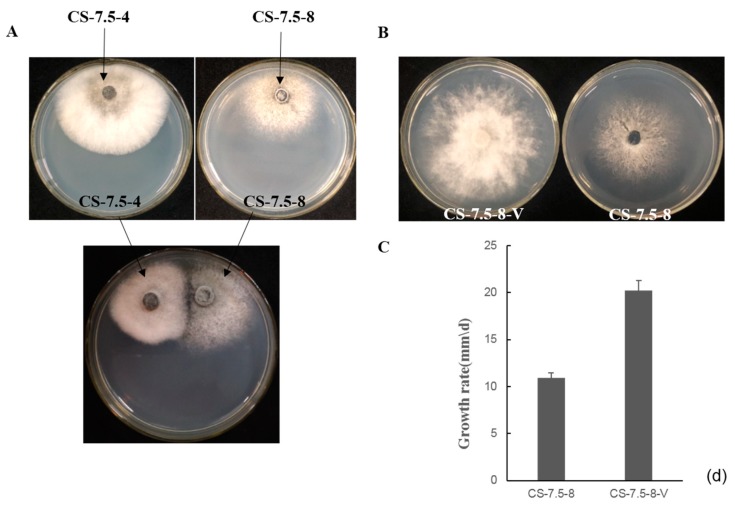
(**A**) Transmission of dsRNA elements from the strain CS-7.5-4 (the donor) to dsRNA-free strain CS-7.5-8 (the recipient) on PDA using pairing culture. Mycelial plugs from the CS-7.5-4/CS-7.5-8 were individually cultured 1 cm apart on a PDA dish. Recipient derivative isolates were obtained by picking mycelial agar plugs from the edge of each colony of the recipient. (**B**) Fungal colony morphology of CS-7.5-8 and the recipient derivative isolate CS-7.5-8-V when cultured on PDA for three days. (**C**) Average growth rates of CS-7.5-8 and CS-7.5-8-V that cultured on PDA for 4 days. Bars indicate standard deviation.

**Figure 8:**
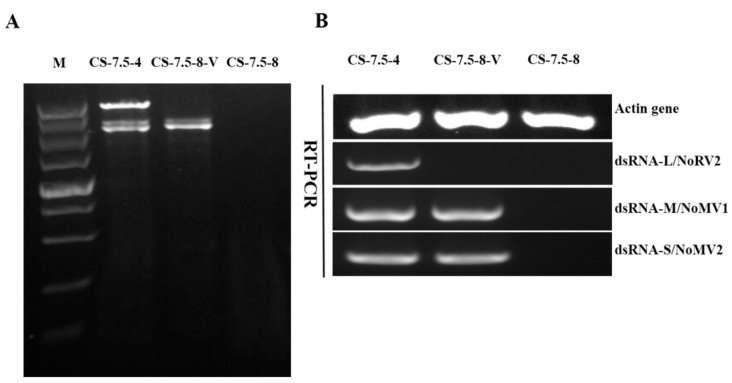
Detection of viruses NoRV2, NoMV1, and NoMV2 in *N. oryzae* strains using dsRNA extraction and RT-PCR methods. **Lane M**, DNA marker (5 kb ladder, TaKaRa). (**A**) Agarose gel electrophoresis of dsRNA extracted from strains of CS-7.5-4, CS-7.5-8, and CS-7.5-8-V. (**B**) RT-PCR assays of the NoRV2, NoMV1, and NoMV2 from strains CS-7.5-4, CS-7.5-8, and CS-7.5-8-V.

The pairing culture experiments between *N. oryzae* strains included three kinds of cultures, as shown in Figure 7A: one double-strains-pairing cultures (CS-7.5-4/CS-7.5-8) and two single-strain cultures (CS-7.5-4 and CS-7.5-8). The dsRNAs containing strain CS-7.5-4 grew rapidly on PDA with thick mycelium, whereas the virus-free strain CS-7.5-8 had normal growth with sparse mycelium. Eight mycelial derivative isolates of strain CS-7.5-8 were obtained from the recipient colonies. One representative derivative isolate CS-7.5-8-V grew rapidly and with more mycelium on PDA when compared to the parental isolate CS-7.5-8. Both dsRNA extraction and RT-PCR analysis showed that only the two mitoviruses (NoMV1 and NoMV2) were positively detected in the CS-7.5-8-V, as well as in CS-7.5-4, but negative in CS-7.5-8 (Figure 8A,B).

The transmission experiments suggested that the two mitoviruses in CS-7.5-4 could be horizontally introduced to CS-7.5-8, and might be responsible for the changes in growth rate and colony morphology of strain CS-7.5-8 (Figure 7B,C).

## 4. Discussion

Although mycoviruses are widespread in almost all major taxonomic fungal groups, the number of currently described mycoviruses is relatively low compared to other viruses infecting plants and animals. As an aim of screening mycovirus that might be a potential biocontrol agent for fungal disease, increasing mycoviruses have been reported [2]. Besides, the discovery of many novel mycoviruses greatly enhanced our understanding of viral diversity, ecology, and evolution. *N. oryzae* is a pathogenic fungus infecting many plants and hosting many mycoviruses that belong to families including Totiviridae, Partitiviridae, Fusariviridae, and other undetermined taxa [21,24,35,36]. In the present study, we characterized three mycoviruses co-infecting a *N. oryzae* strain CS-7.5-4, a victorivirus named NoRV1 and two mitoviruses named NoMV1 and NoMV2, respectively. To our knowledge, this is the first report about the presence of mitoviruses in *N. oryzae*.

NoRV2 was highly similar to the typical victoriviruses in the Totiviridae family such as Hv190SV, BmV1, TcV1, and RnVV1 according to genomic organization and phylogentic analysis. Since NoRV1 was found to be most closely related to Hv190SV, sharing aa identities of 71.6% and 56.7% between their RdRp and CP, respectively. According to the ICTV criterion for species demarcation of victoriviruses, by which 60% aa sequence identity in either the CP or RdRp was used for species distinction [12], NoRV2 should be attributed to a novel member of the genus *Victorivirus* in the Totiviridae family.

Viruses belonging to genus *Mitovirus* of family Narnaviridae are a kind of +ssRNA virus. The simplest unencapsidated mycoviruses have RNA genomes of 2.3–2.7 kb with approximately 62 to 73% A+U content. The mitoviruses could be co-purified with the host mitochondria and used it for protein synthesis [37]. Our results showed that both the genomes of NoMV1 and NoMV2 were predicted to contain a single ORF encoding RdRps with typical motifs of mitoviral RdRps when used the mitochondrial codon usage. The genome of NoMV1 and NoMV2 are AU-rich (NoMV1, 63. 6%; NoMV2, 67.1%). A BLASTp search showed that the RdRp of NoMV1 was most closely related to Sl-aMV5, while NoMV2 was most closely related to AaMV1, with aa identities of 39.1% and 42.3%, respectively. The ICTV species demarcation criterion for mitovirus indicated that the 40% and 90% aa identities are the threshold values to distinguish and affiliate the different and same mitovirus species, respectively [16]. As the aa sequence identities between NoMV1 and Sl-aMV5 or between NoMV1 and AaMV1 were 39.1% and 42.3%, respectively, we considered that both NoMV1 and NoMV2 should be new members of the genus *Mitovirus*.

Sequences of the 5′- and 3′-UTRs of mitochondrial viruses were not conserved. However, the UTRs of the (+) strand of mitoviruses have the potential to fold into stem-loop and hairpin structures [33,38]. In addition, the terminal sequences of some mitoviruses contain the inverted complementarity that could also be fold into panhandle structures, such as the *Ophiostoma mitovirus* 6 [33] and *Sclerotinia sclerotiorum* mitovirus 2 [39]. These secondary structures might be the recognition sites for the RdRp, which harbor important functions in virus replication and translation, and might be the protection architectures acting against the host degrading enzymes [33,38]. The stem-loop structures at both the 5′- and 3′-UTRs of NoMV1 and NoMV2 predicted in this study further stressed the importance of stem-loop structures for mitoviruses and supported the mitochondrial nature of the NoMV1 and NoMV2 genomic RNAs. Although the potential stable panhandle structure has been predicted in NoMV2, it could not be found in NoMV1 due to the lack of the inverted complementary sequences at both ends of the genome. Therefore, it seems that the panhandle structures were not necessary for mitovirus replication or translation. As the NoMV1 and NoMV2 co-infected in *N. oryzae* strain CS-7.5-4 and showed only 33% aa identity within their RdRp, it can be suggested that the two viruses might replicate independently and the difference in secondary structures at the 5′- and 3′-UTRs might participate in the selective recognition of their different RdRps. However, to fully understand the functions of the secondary structures at the UTRs, more studies are needed.

Co-infections by two or more viruses have been reported in many plant pathogenic fungi. For example, multiple co-infections of mitochondrial viruses in the same fungal strains were commonly found, such as these in *Sclerotinia sclerotiorum* and *Ophiostoma novo-ulmi* [26,38,40]. Our study reported the co-infection of two mitoviruses and a victorivirus in the *N. oryzae* strain CS-7.5-4. However, whether there are interactions between the co-infected mitoviruses or between the mitoviruses and victorivirus is not known. As we know, co-infections in some cases might cause interactions between viruses, displayed as synergism or antagonism, where the presence of one virus has impacts on the fitness of other co-infected viruses or bring other effects on the host fungi, such as the co-infection of *Rosellinia necatrix* partitivirus 1 (RnPV1) and *Rosellinia necatrix* megabirnavirus 2 (RnMBV2) in *Rosellinia necatrix.* In these infections, the RnMBV2 could lead to hypovirulence to the host when the RnPV1 is co-infected. However, individual viruses only cause latent infections [41]. In our study, the two mitoviruses co-infected in the *N. oryzae* strain CS-7.5-4 were distantly related to each other, which was considered to be beneficial for stable co-infections. In addition, in general, the mitoviruses and victorivirus were located at different cell compartments of the host fungus, which could also reduce intracellular competition. However, we could not definitely determine whether there was any interaction between these viruses in the host fungus. Therefore, to understand the definite effects and the possible interactions between these viruses and their host fungi, more studies are needed.

Some mitoviral infection associated with hypovirulence of phytopathogenic fungi, such as the *B. cinerea* [42], *S. homo-eocarpa* [43], and *S. sclerotiorum* [38,44,45]. However, most mitoviruses often cause cryptic infections or even the same viral species can lead to variable effects on their host fungus. In our study, in order to clarify if there are any effects on the host fungus, we tried to eliminate these mycoviruses using many methods including hyphal-tip culturing, protoplast regeneration, and chemical and thermal treatments. However, no virus-free or single virus-infected derivative isolates were obtained. Alternatively, we conducted horizontal transmission assays by pairing culture between the virus containing strain CS-7.5-4 and virus-free strain CS-7.5-8. The derivative isolates carrying both NoMV1 and -2 represented by CS-7.5-8V were obtained. Compared with isolate CS-7.5-8, CS-7.5-8V had a greater growing ability, showing a faster mycelial growth rate. In addition, the virus-containing strain CS-7.5-8V (donor) was dual-cultured with the virus-free strain CS-7.5-8 (recipient) (Figures S3,S4). The resultant infected strains also showed a vigorous growth phenotype. This indicated that the two mitoviruses (NoMV1 and -2), at least one of the two viruses, were beneficial for *N. oryzae* growth. Since we have not obtained the derivative isolates containing only the NoRV2 or NoMV1 and NoMV2 alone, we cannot determine the effects of NoRV2 on their host fungus. In addition, which mitovirus was responsible for the beneficial effects to the host fungus needs to be further elucidated.

The efficiency of horizontal transmission of different mycoviruses was variable. As shown in *S.Sclerotiorum*, SsHV2/SX247 was shown to be transmitted between different isolates with a higher efficiency than the other virus SsDRV/SX247 [46]. In another study, Wu et al. [6] indicated that the mitoviruses SnMV1/SsSn-1 and SsMV3/SsSn-1 showed a more efficient transmission than the victorivirus SnVV1. Similarly, in our horizontal transmission experiments, only the two mitoviruses (NoMV1 and NoMV2) were transmitted successfully between *N. oryzae* strains, as no derivative isolates showed infections of NoRV2. Therefore, it seems that mitoviruses, compared with victoriviruses, might spread more efficiently through hyphal anastomosis. However, the mechanism regarding the difference in viral horizontal transmission through hyphal anastomosis is unclear. Polashock et al. [47] reported that mitochondrial movement and recombination was involved in the transmission of mitoviruses mediated via hyphal anastomosis. However, the transmission of victoriviruses during hyphal anastomosis might be complicated, which might be accomplished with cytoplasmic flow, nucleic acid replication, particle assembly and recognition, etc. Thus, more experiments are needed with respect to host antiviral defense and virus multiplication.

## Figures and Tables

**Table 1:** Amino acid sequence identities of the CP and RdRp between NoRV2 and other similar victoriviruses deduced by a BLASTp search.

Virus		CP			RdRp	
	Identity (%)	E Value	Query Cover (%)	Identity (%)	E Value	Query Cover (%)
HvV190S	70.6	0.0	93.9	56.7	0.0	99.6
BmV1	71.3	0.0	93.9	55.9	0.0	99.5
BfTV1	58.7	0.0	98.4	48.2	0.0	99.9
TcV1	61.9	0.0	92.1	47.6	0.0	99.6
SnV1	57.7	0.0	91.9	44.9	0.0	99.4
BbV1	56.7	0.0	92.9	42.1	0.0	99.5
RnV1	59.3	0.0	84.8	41.7	0.0	99.6
AfSV1	53.8	0.0	96.8	39.7	0.0	99.9
UvRV1	51.7	0.0	96.3	40.1	0.0	99.8
UvRVL	51.4	0.0	99.7	40.9	0.0	99.0
SsRV1	48.6	0.0	92.1	41.5	0.0	98.4
SsV1	58.7	0.0	62.7	39.8	2 × 10^−51^	30.8
UvRV5	44.3	5 × 10^−166^	84.8	39.1	5 × 10^−150^	93.5
HmTV1-17	43.6	2 × 10^−15^	83.9	34.9	4 × 10^−149^	97.7
NoRV1	39.1	5 × 10^−126^	91.9	35.1	2 × 10^−115^	91.6
MoV1	39.4	1 × 10^−124^	78.6	37.7	1 × 10^−176^	99.4
MoV3	39.2	2 × 10^−123^	91.5	34.9	5 × 10^−112^	94.9
GaRVL1	39.2	9 × 10^−118^	84.7	36.9	1 × 10^−115^	84.7
AaV1	39.4	2 × 10^−116^	90.9	37.0	5 × 10^−110^	82.9
FpV1	35.6	7 × 10^−116^	93.5	36.0	2 × 10^−105^	75.5

**Table 2:** Results of a BLASTp search of the RdRp amino acid sequences between NoMV1, NoMV2, and other similar mitoviruses.

Virus		NoMV1(RdRp)		Virus		NoMV2(RdRp)	
	Identity (%)	E Value	Query Cover (%)		Identity (%)	E Value	Query Cover (%)
SlaMV5	39.1	2 × 10^−162^	98.1	AaMV1	42.3	0.0	99.3
BcMV3	41.1	7 × 10^−146^	92.4	SlaMV2	41.9	6 × 10^−179^	99.7
FpMV3	37.0	3 × 10^−145^	92.4	AbMV	42.1	5 × 10^−178^	99.4
SsMV28	35.3	8 × 10^−129^	93.2	SlaMV4	52.2	2 × 10^−156^	62.5
RsMV19	56.5	8 × 10^−73^	28.5	FcMV2-1	38.4	1 × 10^−153^	99.3
SsMV29	30.6	2 × 10^−71^	79.9	GaMRV	36.1	9 × 10^−131^	99.3
RsMV20	54.1	8 × 10^−71^	27.5	GMVS1	35.5	1×10^−128^	99.3
RsMV4	32.7	1 × 10^−54^	49.6	SsMV4	38.3	2 × 10^−128^	90.1
RsMV13	26.9	5 × 10^−54^	76.7	BcMV4	40.8	2 × 10^−127^	83.9
MpMV3	31.0	4 × 10^−51^	58.7	SnMV1	40.5	3 × 10^−126^	83.9
SsMV6	31.9	5 × 10^−51^	58.6	FpMV1	37.3	1 × 10^−125^	91.1
ShMV	32.6	3 × 10^−49^	58.1	Omv4	36.7	2 × 10^−116^	89.9

## Supplementary Materials

The following are available online at http://www.mdpi.com/1999-4915/11/1/83/s1, Figure S1: Multiple alignment of the amino acid sequences of RdRp domains of NoRV2 and other similar victorivirus, including HvV190S, BfTV, TcV1, BbV1 and HmTV1-17, Figure S2: Potential predicted secondary structure of the 5′- and 3′-UTRs of NoMV1 (A,B),and NoMV2 (C,D), Figure S3: (A) Horizontal transmission of NoMV1 and NoMV2 from the donor strain CS-7.5-8-V to the recipient strain CS-7.5-8 by pairing culture. Recipient derivative isolates were obtained from edge of the recipient colony. (B) Colony morphology of CS-7.5-8 and the derivative isolate CS-7.5-8-7.5-8-V. (C) Average growth rates of CS-7.5-8 and CS-7.5-8-7.5-8-V when cultured on PDA for 4 days, Figure S4: Detection of NoMV1 and NoMV2 in stains CS-7.5-8-V, CS-7.5-8-7.5-8-V, CS-7.5-8 by dsRNA extraction (A) and RT-PCR (B) using the primers showed in Table S3, Table S1: Information of the primers used for virus detection.

## References

[1] Ghabrial S.A., Suzuki N. (2009). Viruses of plant pathogenic fungi. Annu. Rev. Phytopathol..

[2] Xie J., Jiang D. (2014). New insights into mycoviruses and exploration for the biological control of crop fungal diseases. Annu. Rev. Phytopathol..

[3] Nuss D.L. (1992). Biological control of chestnut blight: An example of virus-mediated attenuation of fungal pathogenesis. Microbiol. Rev..

[4] Yu X., Li B., Fu Y., Jiang D., Ghabrial S.A., Li G., Peng Y., Xie J., Cheng J., Huang J. (2010). A geminivirus-related dna mycovirus that confers hypovirulence to a plant pathogenic fungus. Proc. Natl. Acad. Sci. USA.

[5] Liu L., Xie J., Cheng J., Fu Y., Li G., Yi X., Jiang D. (2014). Fungal negative-stranded RNA virus that is related to bornaviruses and nyaviruses. Proc. Natl. Acad. Sci. USA.

[6] Wu M., Deng Y., Zhou Z., He G., Chen W., Li G. (2016). Characterization of three mycoviruses co-infecting the plant pathogenic fungus sclerotinia nivalis. Virus Res..

[7] Yu L., Sang W., Wu M.D., Zhang J., Yang L., Zhou Y.J., Chen W.D., Li G.Q. (2015). Novel hypovirulence-associated RNA mycovirus in the plant-pathogenic fungus *Botrytis cinerea*: Molecular and biological characterization. Appl. Environ. Microbiol..

[8] Liu H., Fu Y., Xie J., Cheng J., Ghabrial S.A., Li G., Peng Y., Yi X., Jiang D. (2012). Evolutionary genomics of mycovirus-related dsrna viruses reveals cross-family horizontal gene transfer and evolution of diverse viral lineages. BMC Evol. Biol..

[9] Ghabrial S.A., Castón J.R., Jiang D., Nibert M.L., Suzuki N. (2015). 50-Plus years of fungal viruses. Virology.

[10] Ghabrial S.A., Nibert M.L. (2009). Victorivirus, a new genus of fungal viruses in the family Totiviridae. Arch. Virol..

[11] Ghabrial S.A., Dunn S.E., Li H., Xie J., Baker T.S. (2013). Virusesof *Helminthosporium* (*Cochlioblus*) *victoriae*. Adv. Virus Res..

[12] Wickner R.B., Ghabrial S.A., Nibert M.L., Patterson J.L., Wang C.C., King A.M.Q., Adams M.J., Carstens E.B., Lefkowitz E.J. (2011). Family Totiviridae. Virus Taxonomy: Classification and Nomenclature of Viruses, Ninth Report of the International Committee on Taxonomy of Viruses.

[13] Li H., Havens W.M., Nibert M.L., Ghabrial S.A. (2011). RNA sequence determinants of a coupled termination–reinitiation strategy for downstream ORF translation in HvV190S and other victoriviruses (family Totiviridae). J. Virol..

[14] Li H., Havens W.M., Nibert M.L., Ghabrial S.A. (2015). An RNA cassette from Helminthosporium victoriae virus 190S necessary and sufficient for stop/restart translation. Virology.

[15] Wu M., Zhang J., Yang L., Li G. (2016). RNA Mycoviruses and Their Role in Botrytis, Biology. Botrytis—The Fungus, the Pathogen and its Management in Agricultural Systems.

[16] Hillman B.I., Esteban R., King A.M.Q., Adams M.J., Carstens E.B., Lefkowitz E.J. (2011). Family Narnaviridae. Virus Taxonomy: Classification and Nomenclature of Viruses, Ninth Report of the International Committee on Taxonomy of Viruses.

[17] Zoll J., Verweij P.E., Melchers W.J. (2018). Discovery and characterization of novel Aspergillus fumigatus mycoviruses. PLoS ONE.

[18] Kottaloizou I., Coutts R.H. (2017). Studies on the Virome of the Entomopathogenic Fungus Beauveria bassiana Reveal Novel dsRNA Elements and Mild Hypervirulence. PLoS Pathog..

[19] Li L., Pan H., Chen M.Y., Zhang S., Zhong C. (2017). First report of nigrospora oryzae causing brown/black spot disease of Kiwifruit in China. Plant Dis..

[20] Zhang L.X., Li S.S., Tan G.J., Shen J.T., He T. (2012). First report of nigrospora oryzae causing leaf spot of cotton in China. Plant Dis..

[21] Zhai L.F., Liu J., Zhang M.X., Hong N., Wang G.P., Wang L.P. (2013). The first report of leaf spots in aloe vera caused by nigrospora oryzae in China. Plant Dis..

[22] Eken C., Spanbayev A., Tulegenova Z., Yechshzhanov T. (2016). First report of nigrospora oryzae on wheat in Kazakhstan. Plant Dis..

[23] Yu J.X., Zhu J.Z., Wang Y., Zhang C.J., Zhong J., Zhu H.J., Da Gao B., Zhou Q. (2018). Molecular characterization of a putative gammapartitivirus in the phytopathogenic fungus *Nigrospora oryzae*. Arch. Virol..

[24] Zhong J., Zhao S.Q., Li G.F., Pang X.D., Deng X.J., Zhu H.J., Da Gao B., Zhou Q. (2016). A novel fusarivirus isolated from the phytopathogenic fungus nigrospora oryzae. Virus Genes.

[25] Morris T.J., Dodds J.A. (1979). Isolation and analysis of double-stranded rna from virus-infected plant and fungal tissue. Phytopathology.

[26] Xie J., Xiao X., Fu Y., Liu H., Cheng J., Ghabrial S.A., Li G., Jiang D. (2011). A novel mycovirus closely related to hypoviruses that infects the plant pathogenic fungus sclerotinia sclerotiorum. Virology.

[27] Zhong J., Lei X.H., Zhu J.Z., Song G., Zhang Y.D., Chen Y., Da Gao B. (2014). Detection and sequence analysis of two novel co-infecting double-strand rna mycoviruses in *Ustilaginoidea virens*. Arch. Virol..

[28] Larkin M.A., Blackshields G., Brown N.P., Chenna R., McGettigan P.A., McWilliam H., Valentin F., Wallace I.M., Wilm A., Lopez R. (2007). Clustal W and Clustal X version 2.0. Bioinformatics.

[29] Tamura K., Stecher G., Peterson D., Filipski A., Kumar S. (2013). MEGA6: Molecular Evolutionary Genetics Analysis Version 6.0. Mol. Biol. Evol..

[30] Chiba S., Salaipeth L., Lin Y.H., Sasaki A., Kanematsu S., Suzuki N. (2009). A novel bipartite double-stranded rna mycovirus from the white root rot fungus rosellinia necatrix: Molecular and biological characterization, taxonomic considerations, and potential for biological control. J. Virol..

[31] Sasaki A., Kanematsu S., Onoue M., Oyama Y., Yoshida K. (2006). Infection of Rosellinia necatrix with purified viral particles of a member of *Partitiviridae* (RnPV1-W8). Arch. Virol..

[32] Hao F., Ding T., Wu M., Zhang J., Yang L., Chen W., Li G. (2018). Two Novel Hypovirulence-Associated Mycoviruses in the Phytopathogenic Fungus *Botrytis cinerea*: Molecular Characterization and Suppression of Infection Cushion Formation. Viruse.

[33] Hong Y., Dover S.L., Cole T.E., Brasier C.M., Buck K.W. (1999). Multiple Mitochondrial Viruses in an Isolate of the Dutch Elm Disease Fungus Ophiostoma novo-ulmi. Virology.

[34] Mathews D.H. (2004). Using an rna secondary structure partition function to determine confidence in base pairs predicted by free energy minimization. RNA.

[35] Zhou Q., Zhong J., Hu Y., da Gao B. (2016). A novel nonsegmented double-stranded RNA mycovirus identified in the phytopathogenic fungus Nigrospora oryzae shows similarity to partitivirus-like viruses. Arch. Virol..

[36] Zhong J., Zhou Q., Hu Y., Zhu H.J., Da Gao B. (2016). Molecular identification of a novel victorivirus from the phytopathogenic fungus *Nigrospora oryzae*. Virus Genes.

[37] Cole T.E., Hong Y., Brasier C.M., Buck K.W. (2000). Detection of an RNA-dependent RNA polymerase in mitochondria from a mitovirus-infected isolate of the Dutch Elm disease fungus, Ophiostoma novo-ulmi. Virology.

[38] Xie J., Ghabrial S.A. (2012). Molecular characterizations of two mitoviruses co-infecting a hyovirulent isolate of the plant pathogenic fungus Sclerotinia sclerotiorum. Virology.

[39] Khalifa M.E., Pearson M.N. (2014). Molecular characterisation of novel mitoviruses associated with Sclerotinia sclerotiorum. Arch. Virol..

[40] Doherty M., Coutts R.H., Brasier C.M., Buck K.W. (2006). Sequence of RNA-dependent RNA polymerase genes provides evidence for three more distinct mitoviruses in *Ophiostoma novo-ulmi* isolate Ld. Virus Genes.

[41] Sasaki A., Nakamura H., Suzuki N., Kanematsu S. (2016). Characterization of a new megabirnavirus that confers hypovirulence with the aid of a co-infecting partitivirus to the host fungus, Rosellinia necatrix. Virus Res..

[42] Wu M.D., Zhang L., Li G.Q., Jiang D.H., Hou M.S., Huang H.C. (2007). Hypovirulence and double-stranded RNA in *Botrytis cinerea*. Phytopathology.

[43] Deng F., Xu R., Boland G.J. (2003). Hypovirulence-associated double-stranded RNA from Sclerotinia homoeocarpa is conspecific with Ophiostoma novo-ulmi mitovirus 3a-Ld. Phytopathology.

[44] Khalifa M.E., Pearson M.N. (2013). Molecular characterization of three mitoviruses co-infecting a hypovirulent isolate of Sclerotinia sclerotiorum fungus. Virology.

[45] Xu Z., Wu S., Liu L., Cheng J., Fu Y., Jiang D., Xie J. (2015). A mitovirus related to plant mitochondrial gene confers hypovirulence on the phytopathogenic fungus Sclerotinia sclerotiorum. Virus Res..

[46] Hu Z., Wu S., Cheng J., Fu Y., Jiang D., Xie J. (2014). Molecular characterization of two positive-strand RNA viruses co-infecting a hypovirulent strain of Sclerotinia sclerotiorum. Virology.

[47] Polashock J.J., Bedker P.J., Hillman B.I. (1997). Movement of a small mitochondrial double-stranded RNA element of Cryphonectria parasitica: Ascospore inheritance and implications for mitochondrial recombination. Mol. Gen. Genet. MGG.

