# Supplementary material for: A Victorivirus and Two Novel Mitoviruses Co-Infected the Plant Pathogen Nigrospora oryzae
Source: Viruses. 2019 Jan 19;11(1):83. doi: 10.3390/v11010083 (PMC6356909; doi:10.3390/v11010083)
Supplement: Supplementary file 1 [file viruses-11-00083-s001.pdf]

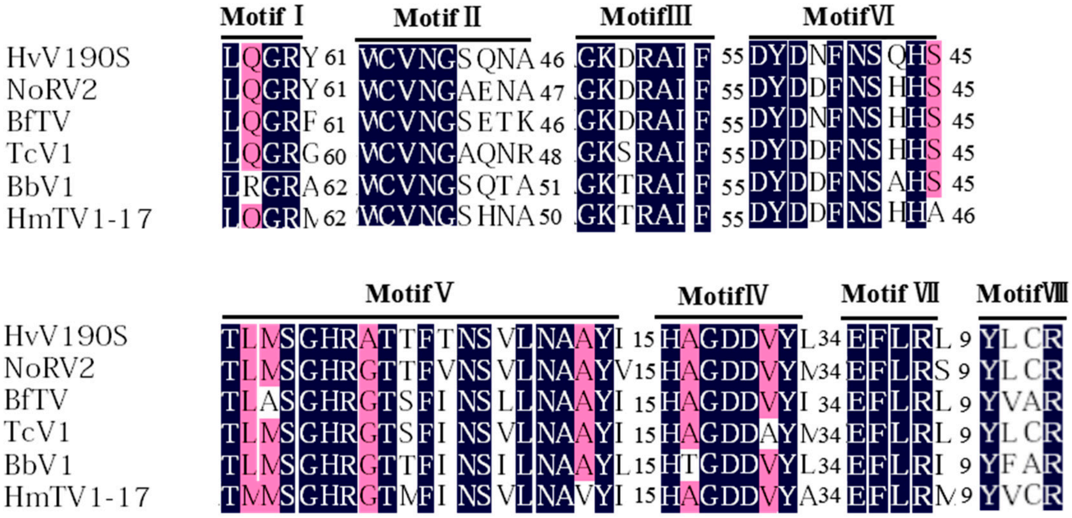

Figure S1. Multiple alignment of the amino acid sequences of RdRp domains of NoRV2 and other similar victorivirus, including HvV190S, BfTV, TcV1, BbV1 and HmTV1-17. Eight motifs, numbered Ⅰ-Ⅷ, that are conserved in the RdRp of these reference viruses were indicated.

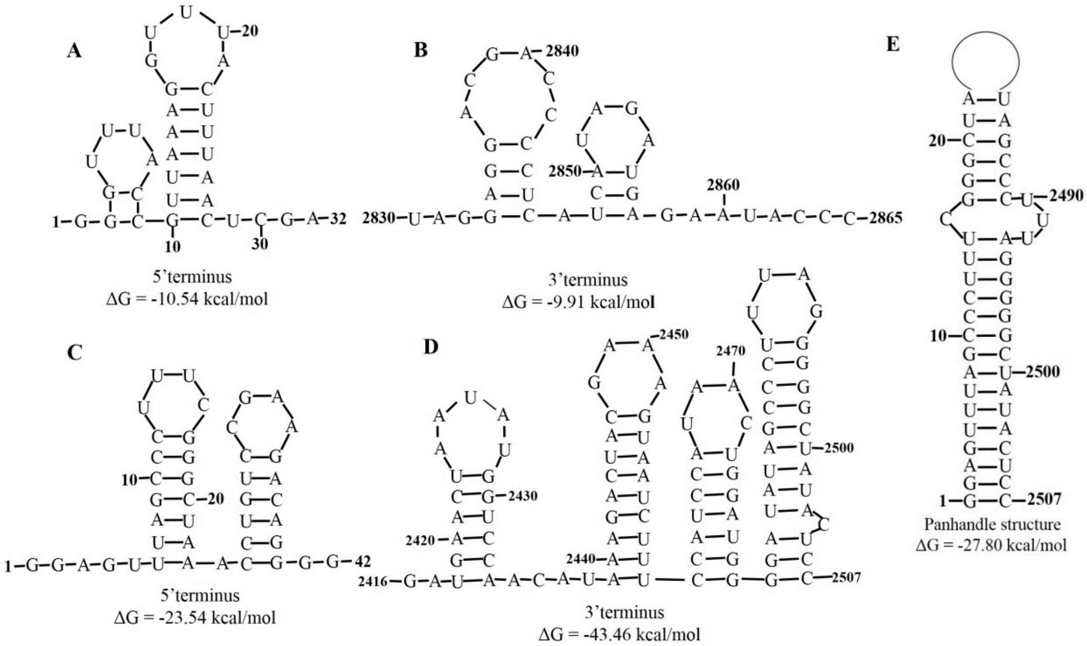

Figure S2. Potential predicted secondary structure of the 5′- and 3′-UTRs of NoMV1 **(A,B)**, and NoMV2 **(C,D)**. Panhandle structure formed by complementary sequences of the 5′- and 3′-terminal sequences of NoMV2 **(E)**. The RNAs were folded and the free energy (ΔG) was estimated using RNA structure software.

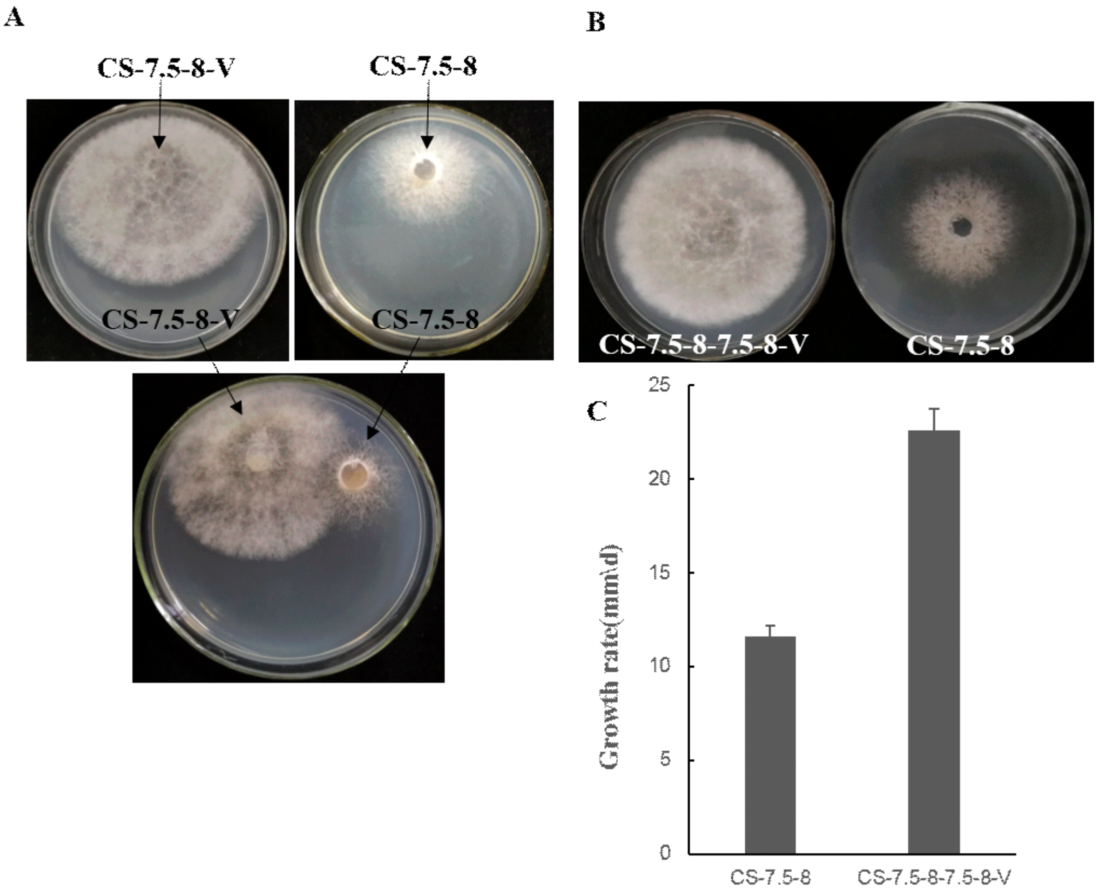

Figure S3. (A) Horizontal transmission of NoMV1 and NoMV2 from the donor strain CS-7.5-8-V to the recipient strain CS-7.5-8 by pairing culture. Recipient derivative isolates were obtained from edge of the recipient colony. (B) Colony morphology of CS-7.5-8 and the derivative isolate CS-7.5-8-7.5-8-V. (C) Average growth rates of CS-7.5-8 and CS-7.5-8-7.5-8-V when cultured on PDA for 4 days.

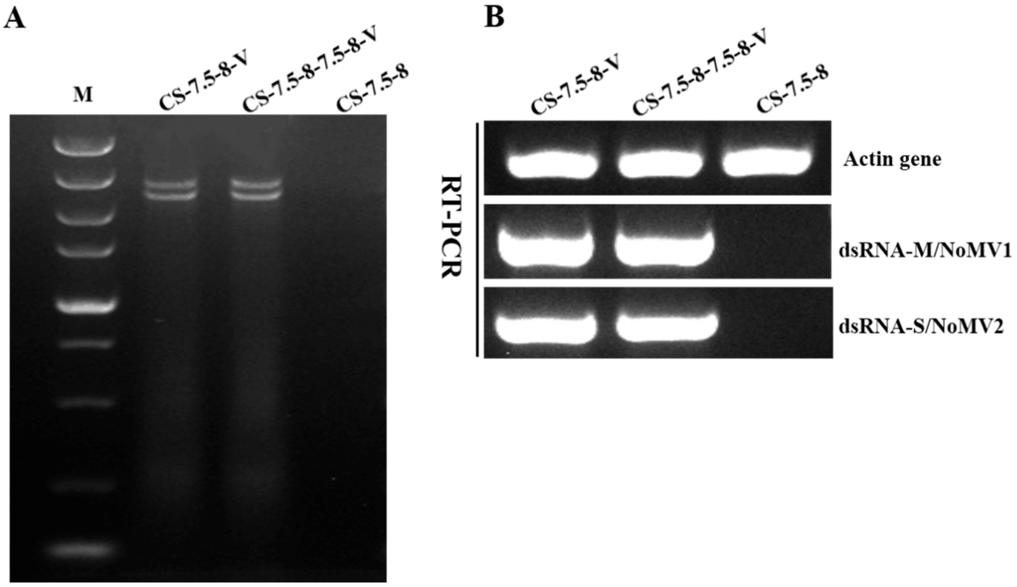

Figure S4. Detection of NoMV1 and NoMV2 in stains CS-7.5-8-V, CS-7.5-8-7.5-8-V, CS-7.5-8 by dsRNA extraction (A) and RT-PCR (B) using the primers showed in Table S3.

Table S1. Information of the primers used for virus detection.

Primer names	Sequences
Actin gene (internal reference)	F:5’-GTCCCCATCTACGAGGGTTTC-3’<br>R: 5’-CGCTCTCAAGACCCAGGACAG-3’
dsRNA-L/NoRV2	F:5’-GACCCTAACACCATTATCCACC-3’<br>R:5’-GCGGCACCACCACCTATT-3’
dsRNA-M/NoMV1	F:5’-TCACTGCTTGCTGCTACACT-3;<br>R:5’-GTCCTCCTACCCTCAACTCC-3’
dsRNA-S/NoMV2	F:5’-CTGTAGGGTTAGCCAAGTTCC-3’<br>R:5’-AAATGAAGGTAGAGTGGGACA-3’
